# Development of Cellulose Nanocrystal (CNC)-Reinforced PLA/PMMA Nanocomposite Coatings for Sustainable Paper-Based Packaging

**DOI:** 10.3390/polym18020175

**Published:** 2026-01-08

**Authors:** Milad Parhizgar, Mohammad Azadfallah, Alireza Kaboorani, Akbar Mastouri, Mariaenrica Frigione

**Affiliations:** 1 Department of Wood and Paper Sciences and Technology, Faculty of Natural Resources, University of Tehran, Shahid Chamran Blvd., Karaj 31585-4314, Iranmastouri_akbar@ut.ac.ir (A.M.); 2 Département des Sciences du Bois et de la Forêt, Faculté de Foresterie, de Géographie et de Géomatique, Université Laval, 2425, Rue de la Terrasse, Quebec, QC G1V 0A6, Canada; alireza.kaboorani.1@ulaval.ca; 3 Department of Engineering for Innovation, University of Salento, Campus Ecotekne, Via per Arnesano, 73100 Lecce, Italy

**Keywords:** poly(methyl methacrylate) (PMMA), polylactic acid (PLA), cellulose nanocrystals (CNCs), cardboard paper, packaging applications, barrier properties, mechanical properties

## Abstract

Driven by environmental concerns, the packaging industry is shifting toward high-performance and bio-based coating alternatives. In this research, poly(methylmethacrylate) (PMMA) and modified cellulose nanocrystal (m-CNC) were employed as reinforcing agents to develop sustainable poly (lactic acid)-based coatings for packaging applications. Various formulations, influenced by polymer matrix blends and m-CNC loadings (1–5%), were prepared using solvent and applied as protective coating on cardboard paper substrates. The grammage of polymeric coatings (CG) on paper was also investigated using various wet film thicknesses (i.e., 150–250 μm). Accordingly, key parameters including water contact angle, thermal behavior, mechanical performances and barrier properties were systematically evaluated to assess the effectiveness of the developed nanocomposite coatings. As a result, nonylphenol ethoxylate surfactant-modified cellulose nanocrystals exhibited good dispersion and stable suspension in chloroform for one hour, improving compatibility and interaction of polymer–CNC fillers. The water vapor permeability (WVP) of PLA-coated papers was significantly reduced by blending PMMA and increasing the content of m-CNC nanofillers. Furthermore, CNC incorporation enhanced the oil resistance of PLA/PMMA-coated cardboard. Pronounced improvements in barrier properties were observed for paper substrates coated with dry coat weight or CG of ~20 g/m^2^ (corresponding to 250 μm wet film thickness). Coatings based on blended polymer—particularly those reinforced with nanofillers—markedly enhanced the hydrophobicity of the cardboard papers. SEM-microscopy confirmed the structural integrity and morphology of the nanocomposite coatings. Regarding mechanical properties, the upgraded nanocomposite copolymer (PLA-75%/PMMA-25%/m-CNC3%) exhibited the highest bending test and tensile strength, achieved on coated papers and free-standing polymeric films, respectively. Based on DSC analysis, the thermal characteristics of the PLA matrix were influenced to some extent by the presence of PMMA and m-CNC. Overall, PLA/PMMA blends with an optimal amount of CNC nanofillers offer promising sustainable coatings for the packaging applications.

## 1. Introduction

The growing environmental concerns associated with the extensive use of petroleum-based plastics in packaging have prompted significant efforts to develop sustainable and biodegradable alternatives. Fossil-based materials, mainly petroleum-derived, are widely used to produce barrier layers that block oxygen, water vapor, grease, and microorganisms [1]. Some common barrier polymers employed in packaging include polypropylene (PP), ethylene vinyl alcohol (EVOH), and polyamide (PA) [2]. Nevertheless, their continued utilization may contribute to resource depletion, rising costs, health risks, and major waste management challenges owing to their non-biodegradable nature [1,2]. Thus, employing sustainable bio-based materials or viable alternatives constitutes a promising pathway toward future advancements.

Polylactic acid (PLA) is a linear aliphatic thermoplastic polyester derived from renewable sources, such as corn starch and sugarcane, offering a sustainable alternative to conventional polymers like polyethylene (PE), polypropylene (PP), polyethylene terephthalate (PET), and polystyrene (PS) [3,4]. Currently, PLA is the second most widely used bio-based plastic worldwide [5]. PLA is immunologically inert and gradually degrades into harmless lactic acid, making it suitable for applications in medicine, packaging, and disposable containers or tableware [4,5]. PLA holds great promise as a sustainable alternative to conventional petroleum-based or non-degradable materials, thanks to its excellent processability, favorable physical–mechanical properties, and high transparency [6,7]. Although bio-based PLA retains many advantages, its inherent brittleness, poor toughness, and low thermal resistance necessitate pre-modification or co-application of reinforcements [5,6,8].

Poly(methyl methacrylate) (PMMA) is a thermoplastic polymer valued for its transparency, tensile strength, and processability [9]. PMMA-based copolymers demonstrate superior mechanical and optical properties, impact resistance, flexibility, and stability against thermal and weathering, along with stress relaxation near the glass transition temperature—features that have driven their broad application, particularly in packaging [10,11]. Therefore, integrating PLA with PMMA represents an innovative approach to enhancing polymer matrix performance. Although some scientific reports exist, the breadth of disciplines and applications highlights that research in this field remains limited and requires further exploration. Therefore, the synergistic use of these polymers under optimal conditions, combined with bio-derived nanofillers, holds great promise for developing durable, high-performance, and eco-friendly films or protective coatings for sustainable packaging.

Cellulose, the most abundant natural biomass, is extracted from plant fibers and possesses a hierarchical structure that allows for the production of nanoscale fillers such as cellulose nanocrystals [12]. Interest in cellulose nanocrystals (CNC) has increased significantly in recent years, especially in materials science and new technologies, due to the industrialization of CNC since 2010 [13]. Due to their high surface area, biocompatibility, low toxicity, and ability to enhance mechanical properties, CNC nanofillers are recognized as effective reinforcing agents in polymeric composite matrices, particularly in PLA-based systems [14,15,16]. Cellulose-based nanofibers are increasingly preferred in packaging applications for their superior barrier properties against oxygen, grease, and mineral oils [17,18]. In this regard, cellulose-based nanofillers (e.g., nanocellulose and CNC) have occasionally been studied and validated in the literature for improving physical–mechanical and barrier properties of coatings applied to wood, paper substrates, and sustainable packaging materials [13,19,20,21]. Although research on these materials has expanded in recent years, the development of high-performance, biocompatible nanocomposite coatings for paper-based packaging remains essential. By the way, the polar nature of cellulose-based fillers necessitates the pre-modification of cellulose nanoparticles prior to use. Surface modification of cellulose nanocrystals not only enhances compatibility within the polymer–filler matrix but also improves the performance of the polymer coating and the resulting nanocomposite [19,22].

This study aims to develop sustainable PLA-based nanocomposite coatings reinforced with poly(methyl methacrylate) (PMMA) and cellulose nanocrystals (CNCs), targeting applications in paper-based packaging. With the aim of improving compatibility, the cellulose nanofillers were first modified. Polymeric formulations containing varying proportions of PLA, PMMA, and modified cellulose nanocrystals (CNCs) were prepared via solvent casting and applied to cardboard substrates at three different polymer loadings. In this regard, key performance parameters such as surface hydrophobicity, bending strength and morphological characteristics (SEM) and barrier properties such as water vapor permeability and oil-repellent efficiency—were systematically evaluated to assess the effectiveness of the developed nanocomposite coatings. In addition, the tensile strength and thermal behavior (via DSC analysis) of the fabricated polymeric films were examined to advance a durable and eco-friendly alternative.

## 2. Materials and Methods

### 2.1. Materials

The poly (lactic acid) (PLA) used in the experiments was obtained from NatureWorks LLC (Minnetonka, MN, USA). Poly(methyl methacrylate) (PMMA) grade of CM-205 was supplied by CHIMEI, Taiwan. Cellulose nanocrystal (CNC) nanoparticles were kindly provided by Forest Products Laboratory in Madison, WI, USA (5.5% suspension). The surfactant, ethoxylated nonylphenol, was received from Paya Resin Company, Isfahan, Iran. The, castor oil was purchased from Farabi Company, Karaj, Iran. Chloroform and fat-soluble red dye (Sudan III) were purchased from Merck, Darmstadt, Germany. A cardboard paper with a basis weight of 180 g per square meter, supplied by Dongguan Xinwu Trade Co. (Dongguan City, Guangdong State, China), was used as the coating substrate.

### 2.2. Surface Modification of CNC Nanoparticles

CNC nanofiller was modified by using ethoxylated nonylphenol (NPE) surfactant, according to a method developed by Fortunati et al. [22]. To modify CNC, a 4:1 (wt/wt) ratio of ethoxylated nonylphenol (NPE) to CNC was used. Initially, 0.5 g of CNC was dispersed in 20 mL of distilled water via ultrasonic stirring for 20 min. Subsequently, 2 g of NPE surfactant was added, and the pH was adjusted to 8 using 1% NaOH. The mixture was stirred at 1400 rpm for 1 h, then transferred to glass containers and freeze-dried for 24 h. The effectiveness of modified cellulose nanocrystal (m-CNC) was assessed through dispersion and stability tests in chloroform solvent. Freeze-dried samples of both modified and unmodified CNC (0.5–1 wt.%) were ultrasonically dispersed in the solvent for 5 min, followed by visual evaluation after 5, 30, and 60 min of storage at room temperature.

### 2.3. Preparation of PLA-Based Nanocomposite Formulations and Coating Process

Formulations based on PLA reinforced with PMMA and m-CNC were prepared using chloroform as the solvent, with treatment details outlined in Table 1. All formulations consistently comprised 8% total ingredients and 92% solvent. For example, for nanocomposite PLA75/PMMA25/m-CNC3: first, 5.82 g of PLA was mixed with 30 mL of chloroform and stirred at 700 rpm for 30 min. Then, 1.94 g of PMMA was mixed with 30 mL of chloroform and stirred at 700 rpm for 30 min. A given amount of m-CNC3 (0.24 g) was dispersed in 32 mL of chloroform under ultrasonic treatment for 5 min. Finally, all three prepared components were combined and mixed for 10 min. Any formulation was then applied as a polymer film on glass or as a coating layer on cardboard paper. In this regard, the formulas listed in Table 1 were applied to pre-prepared and cleaned raw papers (i.e., un-decorated cardboard papers) using a film applicator at 3 different wet thicknesses (i.e., 150, 200, and 250 µm), thereby producing varying dry coat weights. The prepared samples (films or coated papers) were stored at room temperature for one week to dry completely and equilibrate with ambient conditions. The dry coat weight or polymeric coating grammage (CG) on cardboard paper was calculated according to Equation (1).

CG (g/m^2^) = (W_C_ − W_0_)/A

where W_C_ is the weight of the paper after coating (g), W_0_ is the weight of the paper before coating (g), and A is the area of the coated paper (m^2^).

### 2.4. Water Vapor Permeability (WVP)

WVP is one of the most important properties of coated products used in packaging [18]. Water vapor transmission rate (WVTR) of cardboards with nanocomposite coatings was measured according to the ASTM E96/E96M-16 [23] test method. For these measurements, three grams of calcium sulfate was placed in a container. The samples of nanocomposites were fixed on top of the container by using paraffin and the container contents were weighed and placed in a desiccator containing saturated solution of potassium sulfate (RH 98%). The cardboard was placed in the container in a way that the coated surface of cardboard was exposed to the air flow. Then, the desiccator was placed in a conditioning room for 48 h and the sample was weighed every hour until the sample reached a constant weigh. In order to calculate WVTR, the curve of weight gain as a function of time was plotted and the slope line of the curve was determined. By dividing the slope of each line by surface of sample exposed to water vapor, WVTR was calculated according to Equation (1). Finally, by using Equation (2), water vapor permeability (WVP) was obtained [24]. Measurements for each treatment were performed in three replicates.

(1) $$WVTR=\frac{\left(\frac{G}{t}\right)}{A}$$

(2) $$WVP=\frac{WVTR}{P(R1-R2)}\times X$$

where WVTR is the water vapor transmission rate, G is weight gain (g), t is time (h), A is area exposed to moisture transfer (m^2^), G/t the slope obtained from a curve of weight gain vs. time, WVP is water vapor permeability, X is the thickness of the samples, P is the vapor pressure of water at 25 °C, R1 and R2, respectively, are the relative humidity (RH) of desiccator containing calcium sulfate and the relative humidity of desiccator containing saturated solution of potassium sulfate.

### 2.5. Oil Barrier Properties

**Figure 1:**
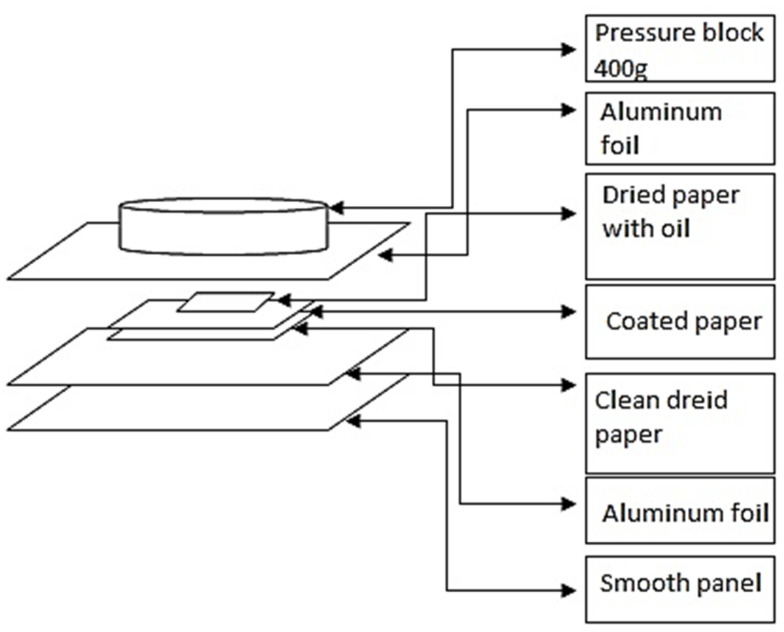
Oil barrier test assembly.

Oil barrier properties were evaluated according to TAPPI T507 standard test methods [25]. For the measurements, castor oil as an edible vegetable oil was employed. Its color was changed to red using Sudan III dye. The method of performing this test is illustrated in Figure 1, where each steps was arranged from bottom to top as follows: 1, smooth panel; 2, aluminum foil of 10 × 10 cm^2^, which is used as a separating plate or spacer; 3, clean dried paper with dimensions of 5 × 5 cm; 4, coated cardboard (upper side); 5, oil-impregnated paper (red colored); 6, aluminum foil. Then, on top of ten columns another pressure block with a weight of 400 g was placed. Finally, the assembled items were kept in an oven at 60 °C for 4 h. The resistance of the tested cardboard to the oil was assessed through the measurement of the area of colored oil spots appeared in the larger dried paper, which was placed under the coated cardboard. Using ImageJ software, Version 1.51, the stained area was quantified, and its ratio to the total surface of the dried paper was calculated as an indicator of oil barrier performance. Measurements for each treatment were performed in three replicates.

### 2.6. Morphological Characteristics

Morphological and topographical analyses of both surface and internal structures of micro- and nano-scale objects were conducted using scanning electron microscopy (SEM, TESCAN MIRA 3, Brno, Czech Republic). Cross-sectional images of the films were obtained from the fracture zones generated during tensile strength testing. For coated cardboard samples, cross-sections were prepared by fracturing the specimens after freezing in liquid nitrogen. Prior to SEM examination at 10 kV, all samples were sputter-coated with a thin layer of gold to enhance conductivity and imaging quality.

### 2.7. Water Contact Angle (WCA)

Water contact angle measurements were used to assess the surface wettability of coated cardboards. The measurements were performed using a dynamic contact angle system (Pocket Goniometer PGX, model 68–76. Fibro System AB, Hägersten, Sweden) with a 5 μL droplet of diluted water applied for 30 s. Three coated samples per treatment were analyzed at room temperature, and the results are reported as mean values.

### 2.8. Mechanical Properties

Mechanical properties were evaluated through two tests: bending resistance and tensile strength. Flexural strength of the coated cardboards with dimensions of 7 mm × 3.2 mm during the bending test was measured using a TABER V-5 tester (Model 150-T, North Tonawanda, NY, USA). Tensile strength of the composite films was evaluated using a Testometric tensile tester equipped with a 50 kN maximum load cell capacity and operated at a crosshead speed (strain rate) of 10 mm/min. For the tensile test, rectangular film specimens with a width of 1 cm and a uniform thickness of approximately 150 μm were prepared and tested using a grip distance of 6 cm. Samples were conditioned at RH = 50% and 23 °C for 7 days prior to conducting the tests. All mechanical tests were performed in triplicate to ensure reproducibility.

### 2.9. Thermal Properties

The thermal behavior of neat PLA and PLA-based polymeric composites reinforced with PMMA or m-CNC was evaluated using differential scanning calorimetry (DSC). Approximately 10 mg of each polymeric sample was analyzed with a NETZSCH 200F3 Maia (NETZSCH-Gerätebau GmbH, Selb, Germany) over a temperature range of 30–200 °C at a heating rate of 10 °C/min under nitrogen atmosphere. Key thermal parameters, including glass transition temperature (Tg), melting temperature (Tm), and crystallization temperature (Tc), were determined to assess the influence of reinforcements on the thermal properties of the polymeric matrices, averaging 3 measurements for each treatment. The degree of crystallinity (Xc) of the PLA phase was calculated from the first heating scan using the following equation:

Xc (%) = [(ΔHm)/(ΔHm^0^ × w)] × 100

where ΔHm is the specific enthalpy of melting obtained from the DSC curves, ΔHm^0^ is the theoretical melting enthalpy of 100% crystalline PLA as reported in the literature (i.e., 93 J/g) [3,4], and w is the mass fraction of PLA in the composite.

## 3. Results and Discussion

### 3.1. Dispersion and Compatibility of Surfactant-Modified CNC

**Figure 2:**
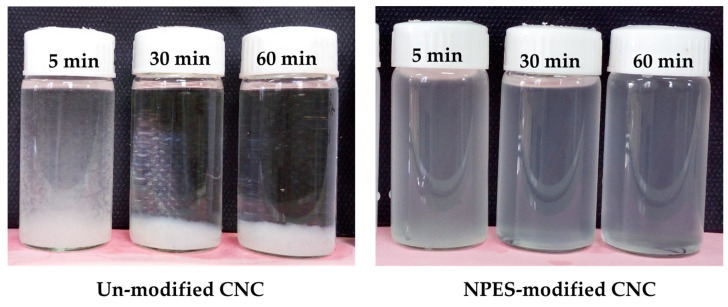
Dispersion and stability of CNC in chloroform medium during storage time from 5 to 60 min: unmodified CNC, nonylphenol ethoxylate (NPE)-modified CNC.

Figure 2 illustrates the dispersion quality and stability of both NPE-modified CNC (i.e., m-CNC) and unmodified nanoparticle (i.e., 0.5–1 wt.%) suspensions in chloroform. The unmodified CNC nanoparticles began settling within 5 min and were fully precipitated after 30 min. In contrast, the m-CNC nanoparticles showed excellent dispersion in chloroform, maintaining a stable suspension for up to 1 h. These observations confirm the effectiveness of the substitution process and the successful modification of the CNC surfaces with ethoxylated nonylphenol surfactant. In this context, acetylation of CNC with acetic-anhydride (A) improved their dispersion in organic solvents and resulted in strong interfacial adhesion between the A-CNC filler and the polymer matrix, which enhanced composite’s mechanical performance and thermal stability [26]. Surface treatment of CNC nanoparticles using electron beam irradiation (EBI) and poly (ethylene oxide) (PEO) has also been reported to enhance nanofiller dispersion within the PLA matrix, thereby improving its mechanical, thermal, and barrier properties [8]. Nonylphenol ethoxylate (NPE), a widely used nonionic surfactant with both hydrophobic (nonylphenol) and hydrophilic (ethoxylate) components, has long been employed in household cleaners, micellar extraction from water, compatibilizer in polymers, and as a surface modifier for cellulose-based nanoparticles [22,27]. As a result, enhanced dispersion not only confirms successful modification of CNC nanofillers but also improves their compatibility with the polymer matrix, thereby contributing to superior copolymer properties.

### 3.2. Polymeric Grammage (Dry Coat Weight)

**Figure 3:**
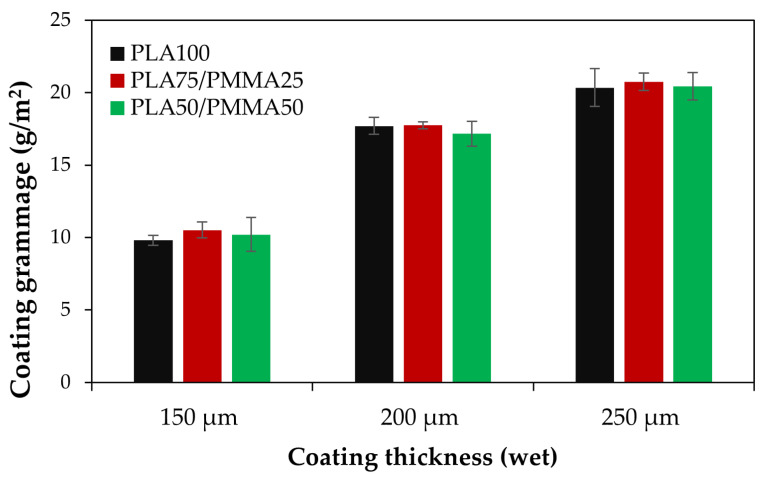
Dry coat weight (coating grammage) affected by various coating wet thicknesses applied on cardboard paper.

Figure 3 illustrates the polymer grammage (i.e., dry coat weight) of cardboard coated with various wet thicknesses and polymer blends. As the wet thickness increased, the polymer loading on the paper also increased; however, no significant differences were observed with the combination of PMMA and PLA. Therefore, CG or dry coat weight of the applied polymer on the cardboard papers varied between ~10 g/m^2^ and ~21 g/m^2^. In general, the grammage of the coating is a key factor influencing the functional properties of coated paper, encompassing hydrophobicity, barrier characteristics, and mechanical strength. However, changes in these properties depend not only on coating thickness but also on the chemical composition of the polymer and its interaction with the substrate [17,20]. Previous studies have reported that increasing the coating thickness to 30 μm on cardboard enhances hydrophobicity and tensile strength, while reducing thermal conductivity and printability [20]. Therefore, determining and applying the optimal grammage is a critical consideration.

On the other hand, various solvents are used to prepare these types of formulations which are eventually evaporated slowly after the casting process, among which chloroform are common suitable solvents for polylactic acid [16,18]. Therefore, these solvents do not pose many limitations and are mostly used to determine the optimal performance and properties of the formulations and the resulting coating on a laboratory scale. Furthermore, these materials (polylactic acid and methyl methacrylate) can also be processed by extrusion coating in industrial applications [17].

### 3.3. Barrier Properties

#### 3.3.1. Results of Water Vapor Permeability (WVP) Tests

The water vapor transmission rate (WVTR) and water vapor permeability (WVP) values for coated cardboard are presented in Table 2. While the WVP value of uncoated cardboard was 4.43 × 10^−6^, the application of protective coatings significantly reduced its water vapor permeability. Incorporating PMMA into the PLA formulation led to a relative decrease in the WVP value. Increasing the m-CNC content consistently reduced water vapor permeability (WVP) across all PLA/PMMA formulations, regardless of the polymer ratio in the nanocomposite composition. Hence, for coatings with wet thickness of 150 µm, the lowest WVP was obtained for the PLA50/PMMA 50/m-CNC3 treatment. The wet and resulting dry thickness and polymeric grammage applied to the paper significantly influenced its water vapor permeability. However, the chemical nature and molecular characteristics of the polymer or its resulting blend are likely to strongly influence the barrier properties. Therefore, the lowest WVP among all formulations was observed in the coated cardboard with a wet thickness of 250 µm (i.e., more coatings grammage), corresponding to treatment PLA50/PMMA 50/m-CNC5. A similar dependence of water vapor permeability on coating thickness has also been reported by other researchers [28]. Enhancing the water-repellent properties of films and coatings can significantly extend the shelf life of food products by reducing moisture transfer and preserving product quality. Water vapor permeability is influenced by several factors such as matrix density, interaction between the blended polymers, particle shape or size, porosity, crystallinity, and film thickness [29,30]. According to previous studies [18], both low density polyethylene (LDPE) and PLA demonstrate water vapor barrier properties, making them widely used materials in packaging applications. On the other hand, the hydrophobic nature of PMMA [31] contributes to the lower water vapor permeability of PLA/PMMA coatings compared to pure PLA-based ones. Kaboorani et al. [32] and Gray et al. [15] also reported that the incorporation of CNC into polymeric composites (i.e., LDPE, thermoplastic starch and wood coatings) reduces its WVP. In fact, CNC nanofilllers have been shown to create a tortuous path within the polymer matrix, which hinders the diffusion of water vapor through the material [33,34].

#### 3.3.2. Oil-Repellent Efficiency

**Figure 4:**
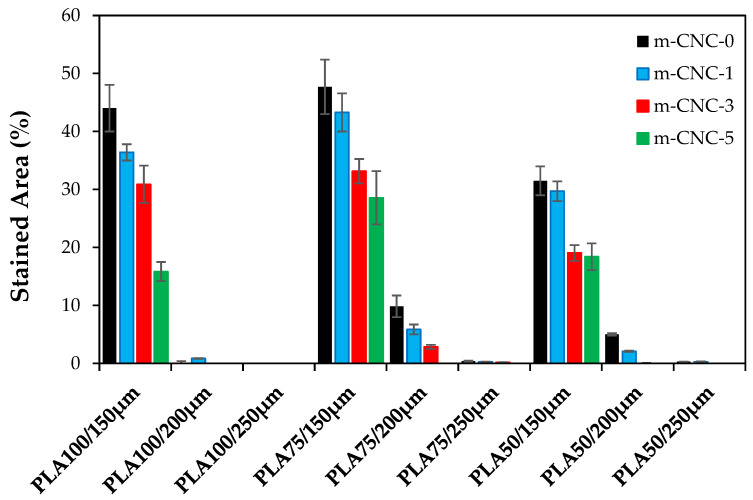
Oil resistance of cardboard with PMMA/PLA/m-CNC nanocomposite coatings.

**Figure 5:**
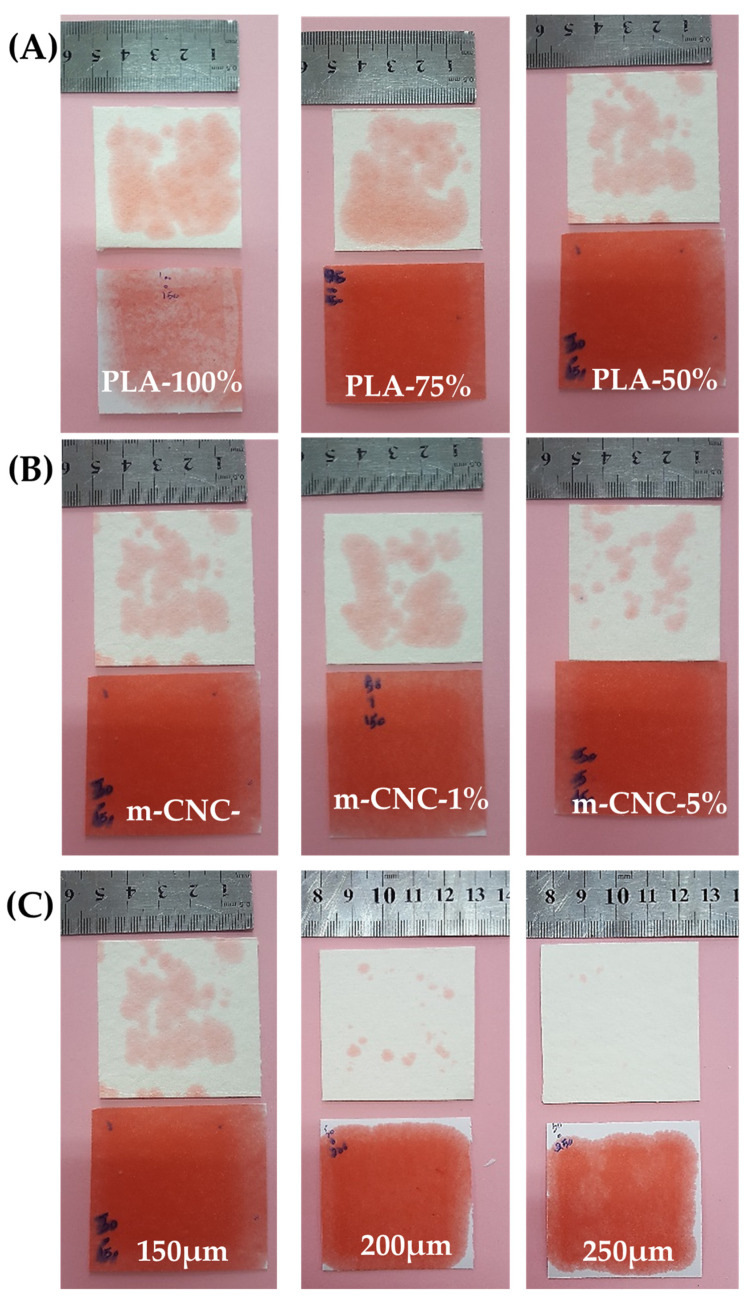
Visual comparison of oil-barrier behavior in PLA/PMMA/m-CNC coatings on cardboard papers: (**A**) effect of PLA-to-PMMA ratio, and influence of (**B**) m-CNC content and (**C**) coating thickness in PLA50/PMMA50 formulation.

The results and images of measuring oil repellency of cardboards coated with PLA/PMMA/m-CNC are shown in Figure 4 and Figure 5, respectively. Regarding copolymer incorporation, variations in the PMMA to PLA ratio did not significantly influence the anti-oil performance of the coated cardboards. The loading m-CNC in the PLA/PMMA formulations significantly influenced the oil repellency of the coated cardboard, i.e., higher m-CNC contents resulted in further improvement in oil barrier performance. Coating thickness exhibited an outstandingly greater influence on the oil barrier properties of the cardboard paper. Thus, paper specimens coated with 250 µm wet film exhibited complete resistance to oil penetration, as indicated by the absence of visible oil stains (Figure 4). Resistance to grease and oil penetration is a key consideration in packaging design, particularly for food products such as butter, vegetable oils, and meat, which are prone to lipid migration. In other words, packaging intended for food contact applications must effectively prevent the migration of mineral oils from printing inks into the interior of the packaging. PLA is a potential candidate that exhibits reasonably high resistance to water vapor transmission, but provides relatively poor barrier properties against oxygen [18]. Although the potential of PLA polymer is evident, its limitations underscore the need for further enhancement to optimize its performance for practical applications. It has been reported that cellulose-based nanofibers (i.e., CNC, CNF) are increasingly favored for packaging applications due to their excellent barrier properties against oxygen, grease, and oils [13,17,18]. In fact, PMMA/PLA/m-CNC composite formed interlocked networks on the surface of cardboard which blocked the passage of oil. Aulin et al. [35] reported that the enhanced barrier properties of micro- and nano-fibrillated cellulose-based (CNF, CMF) films against oxygen and oil, resulting from reduced permeability, are attributed to an increased tortuous path for oxygen diffusion. In other words, the decrease in oxygen permeability with increasing film thickness is attributed to non-interconnected pores and the impermeable crystalline regions of cellulose-based films [35], both of which hinder diffusion and penetration.

### 3.4. Scanning Electron Microscopy (SEM)

**Figure 6:**
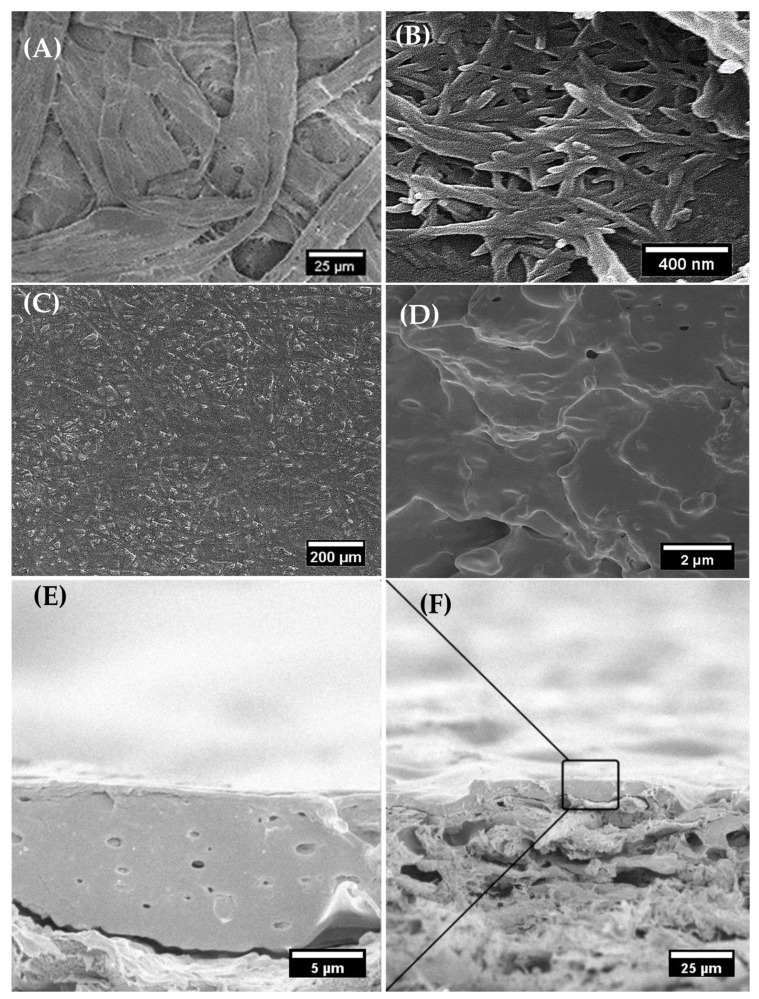
The SEM images of PMMA /PLA/m-CNC-based composites and coated paper: (**A**) uncoated cardboard paper, (**B**) surfactant modified CNC, (**C**) PLA75/PMMA25/m-CNC3 coated cardboard and cross-section images of PLA75/PMMA25/m-CNC3 (**D**) film and (**E**,**F**) applied coating on cardboard paper.

In Figure 6, the SEM images related to composite coatings and cardboard paper are reported. Morphological analysis clearly revealed that the nanocomposite coating of PLA/PMA/m-CNC filled and covered the porous fiber network of the cardboard paper, resulting in a smoother surface (Figure 6A,C). Rhim and Kim [28] demonstrated through SEM analysis that PLA coatings yield a smooth surface on paperboard. The morphology of the modified CNC is also observable in Figure 6B. The cohesive structure of the polymer matrix, i.e., PLA, PMMA, and m-CNC, with no presence of phase separation, demonstrates optimal dispersion and material compatibility (Figure 6D), contributing to the enhanced performance of the nanocomposite coating. Cross-sectional SEM images of the coated cardboard paper with a wet thickness of 200 μm revealed its morphology and a dry coating layer of approximately 12 ± 3 μm on the paper-based substrate (Figure 6E,F).

### 3.5. Results of Water Contact Angle (WCA) Tests

**Figure 7:**
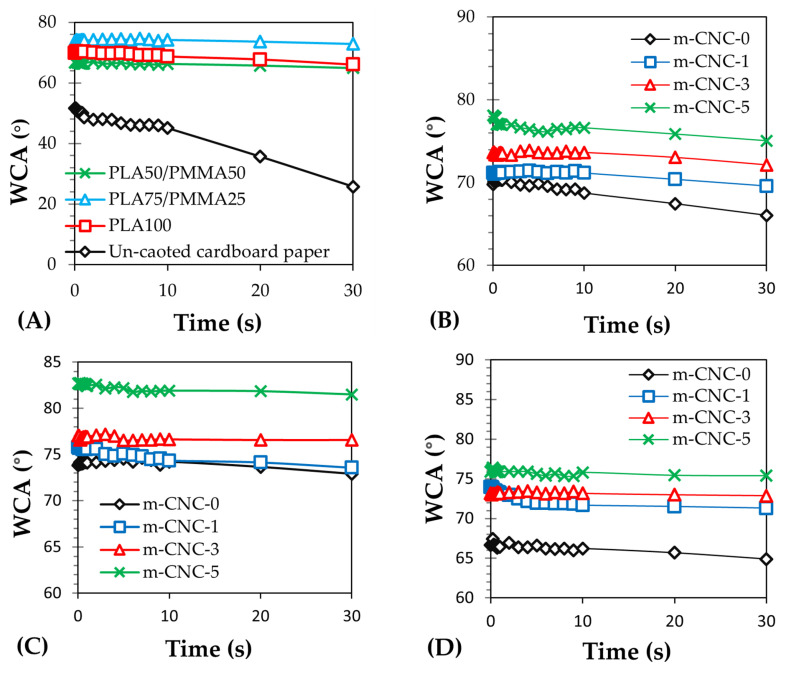
Water contact angle (WCA) of PLA/PMMA-based coatings (wet thickness: 200 µm) applied on cardboard papers: (**A**) effect of PLA-to-PMMA ratio compare to un-coated sample, influence of m-CNC content in (**B**) PLA100, (**C**) PLA75/PMMA25, (**D**) PLA50/PMMA50 formulations.

**Figure 8:**
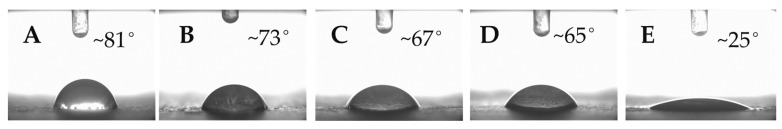
Visual comparison of WCA on composite coatings (wet thickness:200 µm) applied on cardboard papers after 30 s of dripping: (**A**) PLA75/PMMA25/m-CNC-5, (**B**) PLA75/PMMA25, (**C**) PLA100, (**D**) PLA50/PMMA50, (**E**) un-coated sample.

Figure 7 presents the water contact angle measurements of cardboard papers coated with PLA/PMMA/m-CNC-based composite polymers at a wet film thickness of 200 μm. Additionally, images related to the surface wettability performance are presented in Figure 8. Overall, the coating of the paper reduced its wettability and increased the surface WCA, indicating enhanced hydrophobicity (Figure 6A). Incorporation of PMMA into the PLA matrix did not significantly alter the WCA, although the highest hydrophobicity was observed in PLA75/PMMA25-based treatment. Addition of CNC reinforcements to the formulation of coatings increased WCA, especially at 5% content. Finally, the highest WCA (i.e., 81.5°) and the lowest WCA (i.e., 25.5°) after 30 s of dripping were obtained for PLA75/PMMA25-CNCs5 and the uncoated cardboard papers, respectively. Surface wettability is governed by two key factors: morphological characteristics and the chemical nature of the materials surface [36,37,38]. In this regard, the high WCA observed on nanocomposite coatings with elevated nanofiller content may be attributed to increased surface roughness, likely caused by the incorporation of 5% m-CNC. The enhanced water repellency of nanocomposite coatings applied to cardboard substrates can be also attributed to the inherent hydrophobic nature of PLA [28] and PMMA, along with surface topography modifications and improved water barrier properties.

### 3.6. Results of Mechanical Tests

#### 3.6.1. Bending Resistance of Coated Cardboard Papers

**Figure 9:**
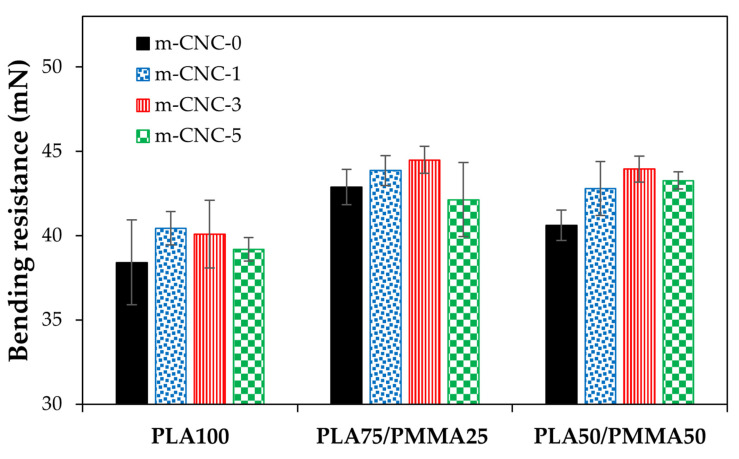
Bending resistance of cardboard papers decorated with PLA/PMMA nanocomposite coatings at 150 µm wet thickness.

**Figure 10:**
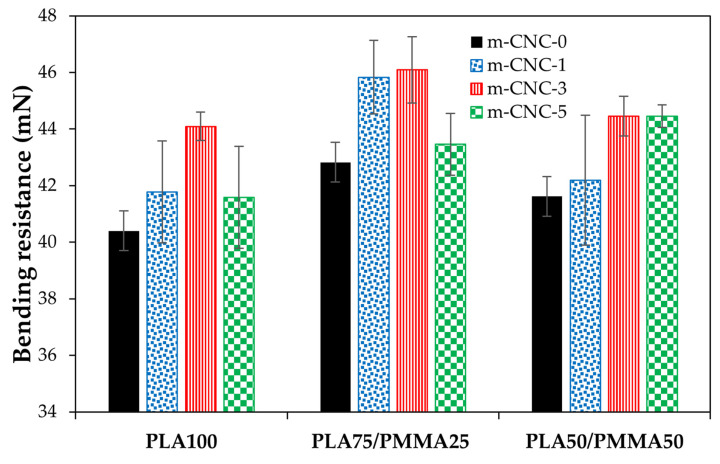
Bending resistance of cardboard papers decorated with PLA/PMMA nanocomposite coatings at 200 µm wet thickness.

**Figure 11:**
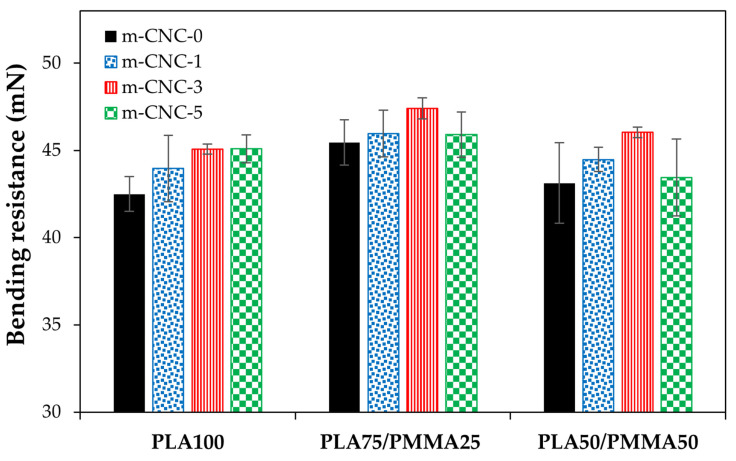
Bending resistance of cardboard papers decorated with PLA/PMMA nanocomposite coatings at 250 µm wet thickness.

The results of bending resistance for cardboard decorated with PLA/PMMA/CNCs-based coatings at different wet film thicknesses are illustrated in Figure 9,  Figure 10 and Figure 11. While the bending resistance of the uncoated cardboard was ~39 mN, the application of various coatings, particularly those with blend polymers, resulted in a notable improvement in its mechanical properties. A relative increase in the bending resistance of coated papers was observed at higher wet film thicknesses. Gicquel et al. [13] reported that thickness of coating film affects the bending strength of coated papers. An improvement in the bending resistance of the resulting copolymers was observed upon incorporating PMMA into PLA polymer across all applied film thicknesses, with the PLA75/PMMA25 blend showing the most pronounced enhancement. The incorporation of modified cellulose nanocrystals (i.e., m-CNC) into the PLA/PMMA copolymer also significantly enhanced the bending resistance of the coated cardboards. However, in most cases, the formulation containing 5% m-CNC exhibited either a declining trend or the least improvement in mechanical performance. Finally, the cardboard papers coated with treatment PLA75/PMMA25/m-CNC3 exhibited the highest flexural resistance in the bending test. In fact, flexural properties reflect a material’s ability to resist fracture when subjected to bending forces. As reported by ref. [11], PMMA exhibits higher flexural strength than PLA. It has also been reported that the impact and flexural strengths of plasticized poly vinyl chloride (PVC)/PLA blends increase with the addition of 10% PMMA to the polymeric phase, which is attributed to improved interfacial interactions [39]. Trivedi and Gupta [40] showed that adding cellulose nanocrystals (CNC) to PLA-based bio-nanocomposites significantly improves flexural and tensile properties at optimal concentrations, due to enhanced interfacial bonding and structural reinforcement.

#### 3.6.2. Tensile Strength of Nanocomposite Films

**Figure 12:**
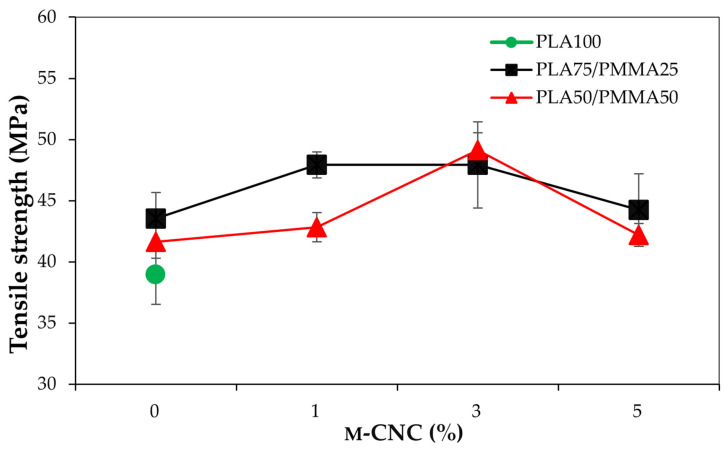
Tensile strength of PLA-based polymeric films influenced by the PMMA and modified CNC contents.

Figure 12 presents the tensile strength results of PLA-based films influenced by the PMMA and m-CNC contents. Polymeric blend films exhibited better tensile performance compared to pure PLA film. In a different trend across, the addition of m-CNC nano-reinforcements enhanced the tensile strength of both PLA/PMMA blends; however, a negative effect was observed at a 5% m-CNC loading. Ultimately, the highest tensile strength (~48–49 MPa) was observed in treatments PLA50/PMMA50/m-CNC3 and PLA75/PMMA25/CNCs1. It has been reported that tensile strength of PLA-based nanocomposites enhanced by approximately 30% compared to neat PLA by adding PMMA-modified cellulose nanofibrils (P-CNF), due to improved interfacial compatibility [41]. Furthermore, modification of CNF with PMMA increases hydrophobicity and also prevents fibril collapse during drying. On the other hand, the high specific surface area of nanofillers probably contributes to increasing the mechanical strength of composite films [42]. Moreover, the limited mechanical enhancement observed at 5% m-CNC loading may be due to poor dispersion and integration within the polymer matrix, suggesting that concentrations ≤ 3% are optimal for effective reinforcement in PLA/MMA blends.

### 3.7. DSC Analysis

**Figure 13:**
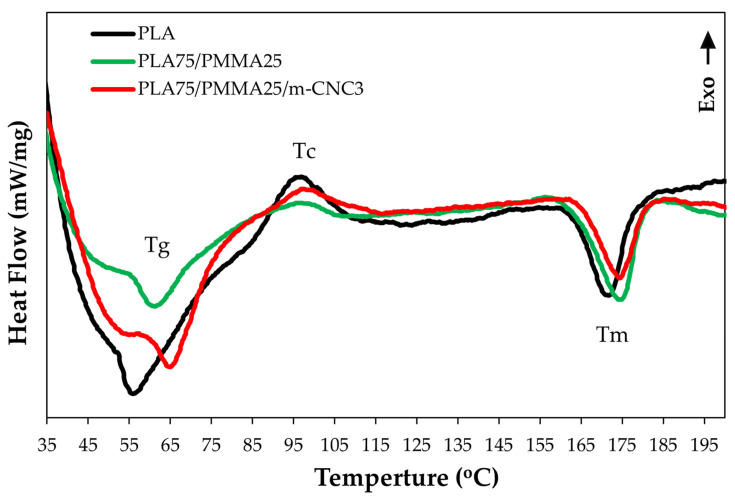
DSC curve of PLA-based nanocomposite copolymers influenced by PMMA and m-CNC fillers.

The DSC curves and corresponding thermal parameters of neat PLA and its reinforced elite blends are presented in Figure 13 and summarized in Table 3. The thermograms shown are from the first heating scan, reflecting the thermal behavior of the samples in their as-prepared state. During heating of the polymer matrices, three signals were observed. The first, between 55 °C and 70 °C, corresponds to the glass transition temperature (Tg) of PLA, partly overlapped by an endothermic peak. The limitations of the DSC instrument used for the analysis did not allow the test to be started at temperatures lower than ambient (i.e., 30 °C).

The endothermic peak superimposed on Tg is attributed to physical aging, an enthalpic relaxation process previously reported for PLA [43]. Adding PMMA and 3% m-CNC nanofillers increased the Tg of PLA, likely reducing chain flexibility. Similar findings were reported by Charasseangpaisarn et al. [11] and Liu et al. [44]. The large surface area of nanoparticles promotes chain entanglement, restricting mobility and raising Tg, while also affecting hydrophobicity and mechanical properties [45]. In addition, hydroxyl groups in cellulose nanomaterials interact with ester groups (COOCH_3_) in PMMA, limiting chain rotation and enhancing the composite’s thermal properties [46,47].

The second signal, observed in the temperature range of 95–100 °C, is attributed to PLA crystallization (Tc). It is likely that PLA, alone or with PMMA, was not slowly cooled, thus remaining amorphous below its crystallization temperature [43]. Thus, the energy supplied to the PLA polymer at temperatures above its Tg allowed a reorganization of the polymer chains into a more ordered-crystalline (i.e., energetically favored) structure, corresponding to an (exothermic) crystallization peak. In the presence of cellulose-based nanofillers, a slight increase in the crystallization temperature (Tc) of PLA was observed with respect to the pure polymer. It is likely due to the formation of a cellulose network, which restricts polymer chain mobility and promotes crystallization—ultimately enhancing mechanical and other properties [41,48]. However, the calculated crystallinity percent (Xc) of the PLA specimens was increased, specially by the composition of PMMA to PLA. The relative decrease in the Xc value of the blend polymer in the presence of nanocrystals indicates that the cellulose-based nanofiller, due to its partial amorphous content, alters the crystallinity of the PLA/PMMA composite during solvent casting, which has also been reported in [4]. The melting enthalpy (ΔHm) also increased for the hybrid laser but decreased with the presence of nanocrystals. The third endothermic peak observed in the DSC thermogram corresponds to the melting process of the PLA phase, characterized by a peak temperature (Tm) centered around 170 °C [49]. Given that the Tm value of PMMA alone is approximately 184 °C [50], it can be inferred that the PLA/PMMA blend exhibits a slightly higher melting point compared to pure PLA. A study by Charasseangpaisarn et al. [11] reported that variations in melting temperature (Tm) can influence the flexural properties of the material. Overall, thermal analysis shows that, beyond enhanced thermal stability of PLA, the composite formulation (PLA50/PMMA50/m-CNC3) also improves its mechanical and physical performance.

## 4. Conclusions

With regard to reducing environmental concerns and advancing high-performance alternatives, PMMA and modified CNC nanoparticles were employed as reinforcing agents to develop sustainable poly (lactic acid)-based nanocomposite coatings for packaging applications. Cellulose nanocrystal (CNC) nanoparticles modified with nonylphenol ethoxylate surfactants exhibited good dispersion and stable suspension in chloroform, confirming the effectiveness of the surface modification process. The water vapor permeability of PLA-based coatings on paper substrates was significantly reduced by the incorporation of PMMA and by increasing the content of m-CNC nanofillers. Additionally, m-CNC loading enhanced the oil-repellent efficiency of the PLA/PMMA-coated cardboard papers. Increasing coating grammage (i.e., dry coat weight) further enhanced performance, with a wet film thickness of 250 µm yielding the highest barrier properties. The protective coating of PLA/PMMA copolymer blends also improved the hydrophobicity of the papers, particularly when containing 5% m-CNC. Mechanical properties (i.e., bending test of coated papers and their film’s tensile strength) were superior for PLA75%/PMMA25% copolymer, especially in the presence of 3% m-CNC reinforcements. Furthermore, the thermal behavior of the PLA matrix was influenced to some extent by the presence of PMMA and modified CNC nanoparticles. Therefore, nanocomposite blend coatings containing an optimal level of CNC nanofillers, with advanced thermal, barrier, and mechanical properties, offer a sustainable approach for the paper-based packaging industry. On the other hand, future work may explore industrial extrusion coating methods to ensure scalability, and comparing these approaches would help clarify optimal processing routes.

## Figures and Tables

**Table 1:** Compositions of nanocomposite films and coatings.

Code	PLA (%)	PMMA (%)	m-CNC (%)
PL100	100	0	0
PLA100/m-CNC1	100	0	1
PLA100/m-CNC3	100	0	3
PLA100/m-CNC5	100	0	5
PLA75/PMMA25	75	25	0
PLA75/PMMA25/m-CNC1	75	25	1
PLA75/PMMA25/m-CNC3	75	25	3
PLA75/PMMA25/m-CNC5	75	25	5
PLA50/PMMA50	50	50	0
PLA50/PMMA50/m-CNC1	50	50	1
PLA50/PMMA50/m-CNC3	50	50	3
PLA50/PMMA50/m-CNC5	50	50	5

**Table 2:** Water vapor transmission rate (WVTR) and water vapor permeability (WVP) of coated cardboards.

	WVTR (g/h·m^2^)			WVP (g·m/Pa·s·m^2^)		
Sample	150 µm	200 µm	250 µm	150 µm	200 µm	250 µm
PLA100	12.11	11.83	8.06	2.25 × 10^−6^	2.199 × 10^−6^	1.49 × 10^−6^
PLA100/m-CNC1	10.63	9.81	6.92	1.98 × 10^−6^	1.82 × 10^−6^	1.28 × 10^−6^
PLA100/m-CNC3	9.44	7.63	5.22	1.75 × 10^−6^	1.42 × 10^−6^	0.97 × 10^−6^
PLA100/m-CNC5	9.95	7.20	6.32	1.86 × 10^−6^	1.34 × 10^−6^	1.17 × 10^−6^
PLA75/PMMA 25	10.83	9.86	7.96	2.01 × 10^−6^	1.88 × 10^−6^	1.48 × 10^−6^
PLA75/PMMA 25/m-CNC1	10.27	9.61	7.34	1.90 × 10^−6^	1.78 × 10^−6^	1.36 × 10^−6^
PLA75/PMMA 25/m-CNC3	8.09	6.82	4.68	1.50 × 10^−6^	1.27 × 10^−6^	0.87 × 10^−6^
PLA75/PMMA 25/m-CNC5	7.60	6.37	4.47	1.41 × 10^−6^	1.18 × 10^−6^	0.83 × 10^−6^
PLA50/PMMA 50	10.93	8.05	7.68	2.04 × 10^−6^	1.49 × 10^−6^	1.14 × 10^−6^
PLA50/PMMA 50/m-CNC1	8.92	7.10	4.99	1.66 × 10^−6^	1.31 × 10^−6^	0.93 × 10^−6^
PLA50/PMMA 50/m-CNC3	7.25	5.54	2.90	1.35 × 10^−6^	1.03 × 10^−6^	0.54 × 10^−6^
PLA50/PMMA 50/m-CNC5	7.25	5.53	2.65	1.35 × 10^−6^	1.03 × 10^−6^	0.49 × 10^−6^

**Table 3:** Glass transition temperature (Tg), crystallization temperature (Tc), melting temperature (Tm), melting enthalpy (ΔHm), and degree of crystallinity (Xc) of PLA-based copolymer as affected by PMMA and m-CNC fillers through DSC analysis.

Treatment	Tg (°C)	Tc (°C)	Tm (°C)	ΔHm (J/g)	Xc (%)
PLA 100	56.0 ± 1.1	96.4 ± 0.9	171.6 ± 1.9	11.40	12.26
PLA 75/PMMA25	61.7 ± 0.9	97.7 ± 0.2	174.3 ± 0.8	13.62	19.50
PLA 75/PMMA25/m-CNC3	65.1 ± 2.2	97.9 ± 0.7	174.5 ± 1.5	9.66	13.85

## Data Availability

The original contributions presented in this study are included in the article. Further inquiries can be directed to the corresponding authors.

## Abbreviations

The following abbreviations are used in this manuscript:

CNC	Cellulose nanocrystal
MMAP	poly(methylmethacrylate)
PLA	Poly (lactic acid)
NPE	Nonylphenol ethoxylates
WVP	Water vapor permeability
EVOH	Ethylene vinyl alcohol
PA	Polyamide
PP	Polypropylene
PET	Polyethylene terephthalate
PS	Polystyrene
PE	Polyethylene
